# Qualitatively and quantitatively similar effects of active and passive maternal tobacco smoke exposure on *in utero *mutagenesis at the *HPRT *locus

**DOI:** 10.1186/1471-2431-5-20

**Published:** 2005-06-29

**Authors:** Stephen G Grant

**Affiliations:** 1 Department of Environmental and Occupational Health, Graduate School of Public Health, University of Pittsburgh, Pittsburgh, Pennsylvania 15260, USA

## Abstract

**Background:**

Induced mutagenesis *in utero *is likely to have life-long repercussions for the exposed fetus, affecting survival, birth weight and susceptibility to both childhood and adult-onset diseases, such as cancer. In the general population, such exposures are likely to be a consequence of the lifestyle choices of the parents, with exposure to tobacco smoke one of the most pervasive and easily documented. Previous studies attempting to establish a direct link between active smoking and levels of somatic mutation have largely discounted the effects of passive or secondary exposure, and have produced contradictory results.

**Methods:**

Data from three studies of possible smoking effects on *in utero *mutagenesis at the *HPRT *locus were compiled and reanalyzed, alone and in combination. Where possible, passive exposure to environmental tobacco smoke was considered as a separate category of exposure, rather than being included in the non-smoking controls. Molecular spectra from these studies were reanalyzed after adjustment for reported mutation frequencies from the individual studies and the entire data set.

**Results:**

A series of related studies on mutation at the X-linked *HPRT *locus in human newborn cord blood samples has led to the novel conclusion that only passive maternal exposure to tobacco mutagens has a significant effect on the developing baby. We performed a pooled analysis of the complete data from these studies, at the levels of both induced mutation frequency and the resulting mutational spectrum.

**Conclusion:**

Our analysis reveals a more commonsensical, yet no less cautionary result: both active maternal smoking and secondary maternal exposure produce quantitatively and qualitatively indistinguishable increases in fetal *HPRT *mutation. Further, it appears that this effect is not perceptibly ameliorated if the mother adjusts her behavior (*i.e*. stops smoking) when pregnancy is confirmed, although this conclusion may also be affected by continued passive exposure.

## Background

It has now been unambiguously established that cigarette smoking causes lung and other cancers, and that exposure to secondary tobacco smoke exhaled by smokers also has a causal role in carcinogenesis [1,2]. Carcinogenic smoke metabolites act primarily as genotoxicants by direct DNA adduction as well as by producing oxidative DNA damage. These DNA adducts therefore act as effective biological markers of tobacco smoke carcinogen exposure, integrating differences in metabolic capacity that modify the activation of such chemicals in the body. A subset of these adducts elude the cellular DNA repair systems, which also exhibit interindividual functional variation, persisting through DNA replication to produce mutations [reviewed in [3]]. Thus, induced mutations in surrogate reporter genes can also act as biomarkers of tobacco smoke carcinogenesis, although there is an attenuation of the genotoxic "signal". Induced mutagenesis is therefore more a measure of biological effect than a quantification of exposure [4]. The *HPRT *assay is the most widely applied measure of *in vivo *mutagenesis and has often been used as an intermediate biomarker of biological effect in exposed populations [5,6]. Although most individual studies and meta-analyses have demonstrated a significant induction of *in vivo *mutation in smokers [6,7], there are still exceptions [8-10]. Similarly, there are contradictory studies on the effect of maternal smoking on mutation frequencies in their unborn offspring [11,12].

Maternal tobacco smoking has been associated with premature delivery, low birth weight, deficient lung and neurological function, and increased risk of perinatal mortality [3,13-16]. During differentiation and development, specific cell types are sequentially induced to proliferate, when they become hypersensitive to cytotoxic agents, resulting in the observed dependence of teratological effects upon timing of exposure [17]. Although it has not been proven, there is great concern that the developing fetus might also be hypersensitive to genotoxic agents, producing many oncogenically "initiated" cells which might then expand during the fulfillment of the developmental program. Many studies have demonstrated that tobacco carcinogens cross the placenta [18,19], so that their mutagenic effects might be detected in the offspring. Additionally, in the particular case of the *HPRT *assay, cord blood mutation frequencies (M_*f*_) measured at birth are approximately 10-fold lower than that predicted from age-dependence data in older children and adults, with a correspondingly low variance, such that cord blood *HPRT *mutation measurements should be uniquely sensitive to inductive effects [20].

A number of studies have been undertaken to determine whether maternal "lifestyle" factors influence *in utero *mutagenesis, often specifically targeting smoking as the putative source of genotoxicants. Indeed, two studies from essentially the same laboratories have come to very different conclusions on this question: first, that there was no detectable effect of maternal tobacco smoke exposure on *HPRT *M_*f *_in cord blood [21], and second, that despite the fact that there was no increase in the M_*f *_of children born to mothers who had been exposed to environmental tobacco smoke, there was a significant shift in the mutational spectrum in these children, indicating that different mechanisms of mutation were responsible for their observed M_*f *_[22,23]. We now report a pooled reanalysis of these data, which provides evidence for a more coherent interpretation of these studies. We find that children of active smokers, women who quit smoking when they found they were pregnant and women who were exposed only to secondary smoke during their pregnancy all had similar, significant increases in T-lymphocyte *HPRT *M_*f *_over offspring of women who reported neither active nor secondary exposure. These data provide a rationale for a shift in the *HPRT *mutational spectra in children of mothers passively exposed to environmental tobacco smoke. These data also provide evidence that secondary smoking exposure can have effects indistinguishable from active smoking. This result is discussed in the context of other attempts to document the genotoxic effects of tobacco smoke, and with regard to our own observations in women who attempted to protect their unborn child by quitting smoking during pregnancy.

## Methods

### Data

All *HPRT *assays were performed by the method of O'Neill *et al *[24] in the laboratories of R.J. Albertini, B.A. Finette and colleagues at the University of Vermont. Exposure histories were obtained by questionnaire at postpartum interviews. Women were considered to be passively exposed if they lived or worked in the presence of active smokers. Tobacco carcinogen biomarkers were not specifically determined in the first study [21], although biomarkers of drug abuse were concurrently monitored and corroborated interview data, and in previous studies in the same hospital population serum thiocyanate and cotinine levels corroborated histories of active smoking [25,26]. Tobacco smoke exposure in the second study [22,23] was assessed by measurement of cord blood cotinine levels [27]. The authors identified two subjects whose cotinine levels were not in agreement with their self-reported tobacco smoke exposure group (MFS72 from the passive exposure group and MFS30 from the "quitters" both had cotinine levels characteristic of active smokers) and excluded them from subsequent analysis. In addition, subject MFS99 from the actively smoking group had undetectable cotinine levels, and cotinine levels were not performed on MFS9 from the passively exposed group. These data were analyzed three ways, first retaining these samples in their self-reported categories, in keeping with the assignment of the data from Manchester *et al *[21], second, deleting them from the analysis as per Finette *et al *[22,23], and third, reassigning these samples to the category indicated by their cotinine results.

Data from the study of Manchester *et al *[21] was obtained by pooling their two data sets identified as "University Hospital Colorado" (tobacco smoke exposure was ascertained in only 60 of the 67 subjects analyzed) and "Private Hospital Colorado". Data from the second study is largely that of Finette *et al *[22] supplemented with new subjects MFS3, MFS14, MFS36, MFS83 and MFS89 in the non-smoking, non-passively exposed group, the addition of MFS12 to the passively exposed group (subsequently removed as an outlier), and the adjustment of the M_*f *_of MFS65 in the passively exposed group, all reported in Finette *et al *[23].

Two outliers (defined as having *HPRT *M_*f *_greater than three standard deviations higher than the population) had been previously identified, one in each of these studies. These values both remained outliers in their respective data sets after ln transformation, but only the highest outlier remained significant after pooling the transformed data from the two studies. Except where specifically mentioned in the text, inclusion or exclusion of these subjects did not affect the statistical analyses performed on these populations.

### Statistical analysis

Pairwise analyses were performed on native and ln transformed data using Student's *t *test assuming equal variance from the statistical toolpack of Microsoft Excel. Nonparametric analyses were performed using the Mann-Whitney *U *test available in MiniTab. Except where noted, all three analyses yielded equivalent results regarding significance. Overall smoking effects were evaluated using single factor ANOVA from Excel.

Comparisons of distributions were performed by the chi-square test in MiniTab. Mutations were considered to be independent if they arose uniquely and/or demonstrated a unique rearrangement of the T-cell receptor β and γ genes [28]. In all three mutational spectra studies considered, mutants were derived from a subset of subjects and, in a small minority of cases, multiple clones were analyzed from the same individual. In the McGinness *et al *[29] and Manchester *et al *[21] studies molecular analyses consisted of Southern blotting with a full length human *HPRT *cDNA as probe. In the Finette *et al *[23] study, all but one mutant was defined more completely by sequencing of the base change(s) involved or of the deletion breakpoints.

## Results and discussion

### Study 1

The first set of data was derived from a cohort of 70 newborns born at two hospitals in Denver, Colorado [21]. *HPRT *M_*f *_were determined on cord blood samples using the clonogenic assay. Smoking status was initially separated into three categories: active smokers, active smokers who quit after confirmation of pregnancy (quitters), and non-smokers. In a secondary analysis, non-smokers were then broken out into those likely to have ongoing exposure to environmental tobacco smoke and those actively avoiding passive or secondary smoking exposure. These categories were based on the paradigm that the genotoxic effects of secondary smoke should be intermediate between those of non-smokers with no secondary exposure and active smokers, and their exposure levels and therefore induced mutation frequencies were expected to be closer to those of the non-exposed population than those of the active smokers. Thus, the *HPRT *M_*f *_of the total non-smoking population (with and without evidence of passive exposure, although clearly skewed towards the passively exposed population, which contributed 20 of the 28 subjects in this category) was used as the basis of comparison for the smoking and quitting groups, and neither was found to have a significant induction of *HPRT *mutants. In the present investigation, we have combined these primary and secondary analyses, using only the non-smokers with no evidence of secondary tobacco smoke exposure as the basis of comparison (this was the only category in the original study reported as having a significantly lower M_*f*_). These data are summarized in Table 1, panel *a*.

**Table 1:** *HPRT *M_*f *_in newborns with and without exposure to tobacco smoke metabolites *in utero*

		*HPRT *M_*f *_(× 10^-6^)				
maternal exposure	N	mean ± SD	median	range	P^1,2^	P^2,3^
*a) data from Manchester *et al [21]						
						
unexposed	18	0.76 ± 0.50	0.61	0.14 – 1.9		
passive only	20	1.60 ± 1.43	1.35	0.30 – 5.3	0.021	
quit during pregnancy^4^	4	1.85 ± 1.16	1.60	0.35 – 3.2	0.004	
smoked throughout	27	1.36 ± 0.99	0.98	0.28 – 3.5	0.019	0.012
						
*b) data from Finette *et al [22,23]						
unexposed	26	0.72 ± 0.53	0.52	0.05 – 1.9		
passive only^5^	22	1.18 ± 1.28	0.67	0.10 – 5.1	0.14	
quit during pregnancy	8	0.79 ± 0.46	0.69	0.18 – 1.8	0.51	
smoked throughout	12	0.71 ± 0.51	0.56	0.14 – 1.8	0.73	0.47
						
*c) pooled data*						
						
unexposed	44	0.73 ± 0.51	0.60	0.05 – 1.9		
passive only^5^	42	1.38 ± 1.36	0.87	0.10 – 5.3	0.006	
quit during pregnancy^4^	12	1.27 ± 0.93	0.91	0.18 – 3.2	0.014	
smoked throughout	39	1.16 ± 0.91	0.87	0.14 – 3.5	0.007	0.007

^1^specific exposed group vs. unexposed *HPRT *M_*f *_from single factor ANOVA on ln transformed data

^2^since these four tests were performed simultaneously, to preserve an overall α of 0.05, the threshold for significance of each individual test should be set at *P *= 0.0125, or, if exposure is only tested for an induction of mutation, *P *= 0.025

^3^overall single factor ANOVA on ln transformed data

^4^excluding outlier with *HPRT *M_*f *_of 14.7 × 10^-6^

^5^excluding outlier with *HPRT *M_*f *_of 45.3 × 10^-6^

One outlier was identified in this data set, defined as an individual with an *HPRT *M_*f *_greater than 3 standard deviations higher than the mean for the population. This individual was born to a women who quit smoking during her pregnancy, and had an M_*f *_of 14.7 × 10^-6^, 10-fold higher than the mean of the entire population, 15-fold higher than the median value.

By breaking the "non-smoking" population into those with and without evidence of environmental tobacco smoke exposure, and using those with no evidence of such passive exposure as baseline, we now show both significant effects of tobacco smoke exposure overall in this population, as well as significant inductions in all three exposed categories. Moreover, the *HPRT *M_*f *_in the three exposed populations were not significantly different from one another (pairwise *P *values ranged from 0.09 to 0.75), unless the outlier was included in the analysis, in which case the "quitters" were significantly higher than all three of the other groups.

### Study 2

The second set of data is derived from two related publications [22,23], that were designed as follow-ups to those of McGinniss *et al *[11] and Manchester *et al *[21]. In the former study, newborns in Burlington, Vermont, demonstrated no detectable effect of maternal active smoking on cord blood *HPRT *M_*f*_, although passive exposure was not considered, and therefore might have been a confounding factor. Subjects for the follow-up studies were recruited from the same university-affiliated hospital in Vermont, and had similar *HPRT *M_*f*_. In this study, passive exposure was assessed by interview and ongoing tobacco smoke exposure was estimated by measurement of cotinine levels in the cord blood. In general, these cotinine measurements confirmed the smoking exposure assignments based on the interviews. These data are summarized in Table 1, panel *b*.

This population also contained an outlier, this one in the passively exposed group, with an M_*f *_of 45.3 × 10^-6^, 30-fold higher than the average of the population and 70-fold higher than the median value.

Finette *et al *[22,23] reported on two different but overlapping subsets of these data, and found no evidence of any type of tobacco smoke exposure affecting *HPRT *M_*f*_. Analysis of the entire data set, as summarized in Table 1, panel *b*, confirms these results. Indeed, only if the extreme outlier is included in the analysis is any comparison even close to significant (unexposed vs. passively exposed, *P *= 0.063).

### Pooled data

These two studies examined similarly sized populations, and both failed initially to demonstrate an influence of tobacco smoke exposure on newborn *HPRT *M_*f*_. These two sets of subjects are geographically distinct, and may differ in other ways, but this cannot be assessed from the published data. No other factor was reported to have significantly affected newborn *HPRT *M_*f *_in either study, however. The M_*f *_of the unexposed populations from the two studies are not significantly different from one another (*P *= 0.48), but the combined exposed population from the Colorado population is 1.5-fold higher than the equivalent population from the Vermont studies, which is significant (P < 0.001). This difference has been attributed to maternal environmental and socioeconomic factors, but nothing has been proven. The distribution of samples between the four smoking exposure categories differs significantly between the two studies (*P *= 0.044), with the major disparity being the proportion of active smokers (39% in the Manchester *et al *study [21] vs. 17% in the Finette *et al *studies [22,23], *P *= 0.006). It is tempting to invoke this difference in population distribution to explain the higher overall M_*f *_of the Colorado population (mean 1.32 × 10^-6^, median 0.96 × 10^-6^, range 0.14–5.3 × 10^-6^) than the population from Vermont (mean 0.89 × 10^-6^, median 0.64 × 10^-6^, range 0.05–5.1 × 10^-6^) (*P *= 0.006) when the outliers are not included in the analysis. However, the proportion of all tobacco-exposed individuals (including active smokers, quitters and passively exposed mothers) is not significantly different between the two populations (74% vs. 62%, *P *= 0.66). The *HPRT *M_*f *_for the pooled data set are given in Table 1, panel *c*.

Analysis of the pooled data from these two studies essentially reiterates the results of the reanalysis of the data from Manchester *et al *[21] discussed above: all three groups of tobacco exposed newborns have *HPRT *M_*f *_significantly higher than the unexposed group, and there is no significant difference between the levels of induced mutation amongst the three exposed populations. These data indicate that tobacco smoke exposure *in utero *does induce detectable *HPRT *mutants in the fetus, and that passive maternal exposure has a similar teratogenic effect as active maternal smoking, a finding that is not unprecedented [30].

### *HPRT *molecular spectra

Despite the lack of evidence for a mutagenic effect of tobacco smoke in their newborn cord bloods, Finette *et al *[23] nevertheless examined the molecular spectrum of *HPRT *mutants in two of their subpopulations, those without evidence of any maternal tobacco smoke exposure and those with passive exposure only. The mutations were classified as a) small, intragenic changes, b) gene rearrangements or deletions, or c) exon 2/3 deletions characteristic of illegitimate VDJ recombination (especially in newborn populations [23,31,32]). These data, summarized in Table 2, panel *a*, suggest a shift in the spectrum of the exposed population to significantly higher proportions of both small mutations and deletions attributable to VDJ recombination. Since there was no overall increase in *HPRT *M_*f *_in this population, however, the exposed population also had a compensatory significantly lower proportion of non-VDJ mediated deletions and rearrangements, suggesting a protective effect of tobacco smoke exposure on these types of mutagenic events. We have found that the need to invoke such a protective effect is reduced if these data are put in perspective of the related studies mentioned above [21,23] and if mutation frequencies are used to normalize the distributions.

**Table 2:** *HPRT *mutational spectra in newborns with and without exposure to tobacco smoke metabolites *in utero*

*a) distribution of mutant clones*						
						
maternal exposure	study	total independent mutants	small mutations (%)	deletions, rearrangements (%)	VDJ recombinant deletions (%)	P^1^
						
unexposed	Finette *et al *[23]	30	10 (33)	14 (47)	6 (20)	
mixed	McGinniss *et al *[11]	41	7 (17)	14 (34)	20 (49)	0.039
mixed	Manchester *et al *[21]	38	13 (34)	16 (42)	9 (24)	0.91
passively exposed	Finette *et al *[23]	35	17 (49)	6 (17)	12 (34)	0.036
						
*b) mutation frequencies for three classes of mutants based on individual studies*						
						
maternal exposure	study	overall mean M_*f *_± SD (× 10^-6^)	small mutations M_*f *_(× 10^-6^)	deletions, rearrangements M_*f *_(× 10^-6^)	VDJ recombinant deletions M_*f *_(× 10^-6^)	P^2^
unexposed	Finette *et al *[22,23]	0.72 ± 0.53	0.24	0.34	0.14	
mixed	McGinniss *et al *[11]	0.64 ± 0.40	0.11	0.22	0.31	0.003
mixed	Manchester *et al *[21]	1.32 ± 1.09^3^	0.45	0.56	0.31	< 0.001
passively exposed	Finette *et al *[22,23]	1.18 ± 1.28^4^	0.57	0.20	0.40	0.002
						
*c) mutation frequencies for three classes of mutants based on pooled data*^5^						
						
unexposed	Finette *et al *[22,23]	0.73 ± 0.51	0.24	0.34	0.15	
mixed	McGinniss *et al *[11]	0.99 ± 0.95	0.17	0.34	0.48	0.008
mixed	Manchester *et al *[21]	0.99 ± 0.95^3^	0.34	0.42	0.24	0.037
passively exposed	Finette *et al *[22,23]	1.38 ± 1.36^4^	0.67	0.24	0.47	< 0.001

^1^χ^2^

^2^*t *tests on ln transformed data

^3^excluding outlier with *HPRT *M_*f *_of 14.7 × 10^-6^

^4^excluding outlier with *HPRT *M_*f *_of 45.3 × 10^-6^

^5^for the purposes of this analysis the data of McGinniss *et al *[11] was pooled with that of Manchester *et al *[21] and Finette *et al *[22,23] to yield a single M_*f*_.

Summaries of the *HPRT *mutational spectra generated from the earlier analysis of a newborn population from Vermont [29] and the Colorado UHD population [21] are also presented in Table 2*a*. These spectra were generated from mutants without regard for their potential tobacco smoke exposure, so are classified as "mixed". The population of McGinniss *et al *[11,29] contained only 20% active smokers, however, while the incidence of passive exposure of the remaining 80% of the population was not estimated. 45% of the population reported in Manchester *et al *[21] actively smoked throughout pregnancy, and another 33% reported ongoing exposure to secondary tobacco smoke; only 13% could be considered unexposed. These data might therefore be expected to begin to show the effects of both active cigarette smoking and passive secondary exposure on cord blood *HPRT *mutagenesis, although the power would not be as great as if they were derived only from defined exposed groups.

In adults, active tobacco smoke exposure has been found to increase the frequency and proportion of small base changes at the *HPRT *gene [33], consistent with the known mechanisms of tobacco smoke mutagens and the types of mutations found in oncogenes in smoking-associated cancers [34,35]. Illegitimate VDJ recombination is a mechanism of mutagenesis unique to T- and B-lymphocytes, and is implicated in many of the molecular events associated with leukemia and lymphoma [36,37]. The human *HPRT *gene contains cryptic sites for this DNA splicing event, resulting in the deletion of exons 2 and 3 [31,38], and the occurrence of this type of *HPRT *mutation seems to be associated with the incidence of acute lymphocytic leukemia in children [39]. Elevated levels of illegitimate VDJ recombination have been found in workers occupationally exposed to pesticides and herbicides [40], especially 2,4-dichlorophenoxyacetic acid [41,42] and in cancer patients undergoing chemotherapy [43], particularly with the DNA topoisomerase inhibiting agent etoposide [44,45].

Overall, the two newborn populations from Vermont had indistinguishable *HPRT *M_*f *_(*P *= 0.50), and the total population data from McGinniss *et al *[11] was also consistent with the unexposed group reported by Finette *et al *[22,23] (*P *= 0.51), but the passively exposed group had a significantly higher level of mutation (*P *= 0.013). The distribution of mutants among the three mechanistic classes differed significantly in both cases, however, with the mutants from McGinniss *et al *[29] exhibiting less small mutation and more VDJ recombination-mediated deletion than either group from Finette *et al *[22,23]. The Colorado population had a significantly higher M_*f *_than the unexposed subset of the second Vermont population (*P *= 0.008), but a very similar distribution of mutants (*P *= 0.91). On the other hand, the Colorado population had a similar mutation frequency as the passively exposed subpopulation from this study (*P *= 0.55), but a somewhat different mutant distribution (*P *= 0.068). We believe that these mutational comparisons are of little use unless both frequency and distribution are taken into account at the same time. In Table 2, panels *b *and *c*, the overall *HPRT *M_*f *_from these individual studies and subpopulations (panel *b*), or the M_*f *_generated from our pooled analysis (panel *c*), are used to calculate the frequency of each type of mutant in each population, as was done in Manchester *et al *[21] and Finette *et al *[23].

Expressing the mutational classes as frequencies makes it easier to see the general trends in these studies and their inconsistencies. The frequency of VDJ recombination-mediated deletions is now increased in all exposed populations, and the results from the mixed tobacco smoke populations are consistent with an intermediate level of exposure (remember that even though these populations should contain maternal active smoking exposures, and quitters, the meta-analysis indicated that these should have induced M_*f *_similar to the passively exposed population). The differences in the frequencies of non-VDJ recombination-mediated deletions and rearrangements are diminished under these circumstances. The increase in frequency of small mutations observed in the passively exposed population of Finette *et al *[22,23] is difficult to rationalize with the low levels found in the McGinniss *et al *[29] study, however, the induction in the Manchester *et al *[21] population is again intermediate between those of the two subpopulations from Finette *et al *[22,23]. Significantly, none of the decreases observed in the frequencies of mutational subclasses from the unexposed population of Finette *et al *[23] were themselves statistically significant.

## Discussion

Pooling data from studies applying similar techniques has been shown to be a useful method of investigating subtle effects in molecular epidemiology [46]. The present data was derived from a limited number of studies, however, and may contain unintended bias based on mutation detection methods, study design or uncontrolled confounders. Moreover, we stress that all of this data is based on mutation at a single locus, the X-linked *HPRT *gene, which may not be representative of the entire genome [5].

All cells in the embryo undergo periods of rapid differentiation and proliferation. It has long been postulated that rapidly growing cells are at increased risk of genotoxic damage; this idea is based on the hierarchy of tissues affected by ionizing radiation exposure, the response of tumors to genotoxic chemotherapy, and has been put forward as a way to rationalize hormonal carcinogenesis with the somatic mutational basis of cancer. While *in utero *exposures have been associated with later increases in cancer susceptibility, this research has mostly involved agents that interfere with the differentiation process [47], rather than classical mutagens [48].

## Conclusion

This analysis demonstrates that, despite the conclusions of the original papers presenting the data, both active and passive tobacco smoke exposure *in utero *results in increased fetal mutation at the *HPRT *locus. The observed mutational induction by passive maternal tobacco smoke exposure clarifies the shift in the *HPRT *mutational spectrum previously reported [23], without requiring a complementary protective effect of tobacco smoke on certain types of mutation. The types of mutations observed are consistent with the known mechanisms of tobacco smoke mutagenesis, as well as the unique biochemistry of T-lymphocytes during *in utero *development. The establishment of these *in utero *tobacco smoke effects depended not only on the size of the pooled data set, but also on the judicious selection of a control group, and should abundantly demonstrate the long-term benefits of publishing data in a form that allows for such a re-analysis.

The observation that tobacco smoke mutational effects were not significantly ameliorated by quitting active smoking after the first trimester is troubling. It may well be consistent with the "all-or-none" quality of toxic exposures early in development, although it is doubtful that mutations arising by the mechanism of VDJ recombination-mediated deletion are possible at such an early stage of development. A more probable explanation for the persistent mutational induction observed in the quitters may involve continued passive exposure, since smoking mothers are far more likely to also be exposed to secondary smoke in the home [49]. This question should be directly addressed. We are presently analyzing data from another large set of newborns. In a preliminary report of the first third of this data maternal exposure to alcohol rather than tobacco was associated with higher *HPRT *M_*f *_[50], although there was a shift in the mutational spectrum in the children of smoking mothers consistent with those described here [51].

Overall, these data suggest that further modification of residential and occupational exposures may be necessary to protect the developing fetus from tobacco smoke mutagenesis during pregnancy. If passive exposure does as much damage to the fetus as active smoking, it is imperative that workplace protection be offered to pregnant women, or better, to women who might or intend to become pregnant. This protection must also be provided in the home, where not only the mother, but any other smoking members of the household should be encouraged to quit for the duration of the pregnancy (or longer), or at least should not smoke in the presence of the pregnant woman.

## Abbreviations

HPRT, hypoxanthine-guanine phosphoribosyltransferase; M_*f*_, mutation frequency.

## Competing interests

The author declares that he has no competing interests.

## Authors' contributions

This study was conceived and performed by SGG.

## Pre-publication history

The pre-publication history for this paper can be accessed here:


